# The beta1-adrenergic receptor in the heart

**DOI:** 10.1038/s41420-025-02907-w

**Published:** 2025-12-10

**Authors:** Wenchao Xu, Jie Li, Jie Ju, Min Liu, Wenxu Wang, Min Cheng, Xiaoyun Zhang, Xiaodong Cui, Hao Chen

**Affiliations:** 1 Department of Physiology, School of Basic Medical Sciences, Shandong Second Medical University, Weifang, Shandong China; 2 Weifang Key Laboratory of Basic Research on Chronic Diseases and Stem Cell Therapy, School of Basic Medical Sciences, Shandong Second Medical University, Weifang, Shandong China; 3 Department of Physiology & Pathophysiology, School of Basic Medical Sciences, Shandong Second Medical University, Weifang, Shandong China; 4 Department of neurology, Sunshine Union Hospital, Weifang, Shandong China

**Keywords:** Mechanisms of disease, Cardiovascular diseases, Cell signalling

## Abstract

beta1-adrenergic receptor (β_1_-AR) belongs to G protein-coupled receptors, regulating cardiac physiological and pathological process through complex signaling pathways. Physiologically, the activation of β_1_-AR produces positive chronotropic, positive inotropic and positive dromotropic effects in the heart. However, excessive or sustained activation of β_1_-AR can cause myocardial injury, arrhythmias, and heart failure. The β_1_-AR in the heart exhibits tissue-specific distribution patterns and subcellular localization features adapted to its function within cardiomyocytes. Upon ligand binding, the β_1_-AR undergoes conformational changes and transmits signaling through G protein-dependent pathways (β_1_-AR/Gs and β_1_-AR/Gi) as well as a G protein-independent pathway (β_1_-AR/β-arrestin) to regulate cardiac activity. Subsequently, the β_1_-AR can either dissociate from G protein to undergo desensitization and terminate signal transduction, or it can be endocytosed into the cell, transported to the lysosome to be degraded, or returned to the plasma membrane to continue its function. Additionally, it has been found that β_1_-AR can cause or exacerbate heart disease when abnormal changes occur in its distribution density, localization, and mediated downstream signaling pathways. Therefore, β_1_-AR represents an important pharmacotherapeutic target for the treatment of cardiac diseases. Among the relevant therapeutic agents, β_1_-AR blockers designed specifically against β_1_-AR have evolved to the third generation. This review comprehensively analyzes β_1_-AR from perspectives including its research history, expression, and distribution in the heart, protein structure, signaling pathways, and associations with cardiac diseases.

## Facts

- Under physiological conditions, β_1_-AR is a core protein that regulates cardiac function. In disease states, the expression and function of β_1_-AR undergo dramatic changes. In chronic heart failure, the expression level of β_1_-AR decreases from the normal proportion of 80% to approximately 60%. One of the reasons is the enhanced degradation pathway of the receptor.
- The signaling pathways of β_1_-AR include G protein-dependent and G protein-independent signaling pathways. The G protein-dependent pathways consist of the β_1_-AR/Gs pathway and the β_1_-AR/Gi pathway. The G protein-independent signaling pathway is predominantly the β_1_-AR/β-arrestin signaling pathway.
- β_1_-AR blockers can improve cardiac function by acting on the sympathetic nervous system to inhibit β_1_-AR-mediated signaling pathways.

## Open Questions

- In cardiac diseases, what are the impacts of alterations in the expression level and distribution of β_1_-AR on the heart?
- What are the molecular mechanisms underlying the therapeutic targeting of β_1_-AR in cardiac diseases?
- What are the improved strategies for next-generation β_1_-AR blockers in the treatment of heart diseases?

## Introduction

As a vital organ of the human body, the heart continuously pumps blood, ensuring normal physiological activities. In the intricate regulation of cardiac activities, the function of the sympathetic nerve is of great significance. Excitation of the sympathetic nervous system can release norepinephrine (NE). Subsequently, NE activates the beta-adrenergic receptors (β-ARs) in cardiomyocytes, inducing excitation-contraction coupling in the heart [1]. The heart expresses three types of β-ARs, specifically the beta1-adrenergic receptor (β_1_-AR), the beta2-adrenergic receptor (β_2_-AR), and the beta3-adrenergic receptor (β_3_-AR) [2]. Among these, up to 80% are β_1_-ARs [3]. As the most abundant subtype in the heart, β_1_-ARs are essential for maintaining normal cardiac function.

The signaling pathways of β_1_-AR are complex and diverse. It regulates cardiac function through G protein-dependent pathways (β_1_-AR/Gs and β_1_-AR/Gi) and the non-G-protein pathway (β_1_-AR/β-arrestin). Given the pivotal role of β_1_-AR in cardiac function regulation, it has been used as a well-established therapeutic target for cardiovascular diseases. β_1_-AR blockers competitively bind to the ligand-binding sites of β_1_-AR, antagonizing NE. Due to the lack of intrinsic activity, β_1_-AR blockers prevent the signal transduction of β_1_-AR when bound to the ligand, decreasing the response of the heart to sympathetic stimulation, which in turn leads to the therapeutic effects in cardiovascular diseases.

In this review, we summarize the research history of β_1_-AR, with a particular focus on elucidating its molecular structure and signaling pathways. We delve into the roles played by β_1_-AR in the pathogenesis of various cardiac diseases. Moreover, we discuss the current status of the application and research of β_1_-AR blockers. This review aims to offer a theoretical foundation for the development of therapeutic strategies for cardiac diseases.

## The discovery process of β_1_-AR

The concept of “receptor” first appeared in the early 20th century. In 1905, John Newport Langley analyzed the effects of nicotine and curare on the contraction of striated muscle, discovering that these two drugs acted on the “accessory substance” of the cell membrane to transmit stimuli that determined the biological effects of the cells [4]. In 1908, Paul Ehrlich referred to the special chemical groups in the protoplasm that interacted with drugs as “receptors” [5]. In 1913, Henry Hallett Dale discovered the interaction of epinephrine and ergotoxin in cats with destroyed spinal cords. Epinephrine alone elevates coronary blood pressure, whereas its coadministration with ergotoxin reduces coronary blood pressure. At the time, however, Henry Hallett Dale did not analyze why this phenomenon occurred from a receptor perspective [6]. It was not until 1948 that Raymond Parker Ahlquist first confirmed evidence for the existence of adrenergic receptors and categorized them into alpha and beta types [7]. Subsequently, James Whyte Black developed the first clinically applicable β-AR blockers based on this theory in 1962: propranolol [8]. In 1967, Anthony M. Lands and his colleagues subdivided β-AR into two subtypes, β_1_-AR and β_2_-AR. They also elucidated the differences in the actions of the two subtypes. β_1_-AR is mainly related to cardiac function and manifests itself in enhanced myocardial contractility, while β_2_-AR is primarily associated with bronchodilatation and vasodilatation [9]. In 1974, Bo Ablad proposed the coexistence of β_1_-AR and β_2_-AR subtypes in the human heart when analyzing the effects of selective and nonselective β_1_-AR antagonists on different catecholamines [10]. In 1987, researchers clarified that the human β_1_-AR cDNA is composed of 2400 base pairs through cDNA libraries and named the cDNA of β_1_-AR as ADRB1 [11]. In 1990, it was further determined that the human β_1_-AR gene was localized on 10q24-q26 [12]. Although research on β_1_-AR started early, it was not until 2008 that researchers first analyzed the protein structure of β_1_-AR in turkeys due to the challenges posed by the high activity and low stability of the receptor itself. This is the first protein structure of β_1_-AR [13]. In 2021, researchers resolved the protein structure of the human β_1_-AR [14]. The above research efforts have continued to refine the understanding of β_1_-AR and laid the foundation for β_1_-AR research.

## Expression and distribution of β_1_-AR

### The expression and distribution patterns of β_1_-AR in the heart

In the physiological state, β_1_-AR accounts for approximately 80% of the total cardiac β-AR [3]. Radioligand binding assays [15, 16] and in vivo positron emission tomography experiments [17] have confirmed that the β_1_-AR in the human heart is relatively uniformly distributed in the myocardial tissue. Despite the relatively homogeneous distribution of β-AR, the distribution of the subtypes varies. The distribution density of β_1_-AR in various regions of the heart, from highest to lowest, is as follows: sinoatrial node, ventricles, and atria [3, 18]. Studies have found that the density of β_1_-AR in the sinoatrial node is 4.2 times higher than that in the atrial myocardium [19]. The differential expression of β_1_-AR between the sinoatrial node and the atrium demonstrates its special role in regulating heart rate and rhythm. Approximately 60% of the β-AR in both the right atrial appendage and left ventricle is β_1_-AR [20]. Enrichment of β_1_-AR in specific regions allows the heart to respond more rapidly to the sympathetic nervous system, resulting in positive chronotropic (accelerated heart rate), positive inotropic (enhanced myocardial contraction), and positive dromotropic (accelerated conduction velocity) effects.

The expression level of β_1_-AR is not constant, but is adjusted according to the state of the heart. Analysis of heart tissue from young and aged rats revealed that the mRNA levels of β_1_-AR decreased with age [21] **(**Table 1**)**. A study on cardiac β_1_-AR changes revealed that β_1_-AR decreased more significantly with age in women than in men. A curvilinear relationship between age and receptor density stabilized around age 40 in women, whereas there was no statistically significant difference in the β_1_-AR density in the hearts of men among different age groups. This suggests that age and estrogen may be related to the amount of β_1_-AR expression in the heart [22] **(**Table 1**)**. Additionally, a significant reduction in the number of β_1_-AR has been observed in various cardiac diseases, including dilated cardiomyopathy, aortic valve disease, ischemic cardiomyopathy, mitral valve disease, and tetralogy of Fallot [23]. Radiographic autoradiography showed that the density ratio of β_1_-AR between endocardium and epicardium was 1.0 ± 0.2 in the normal heart but decreased to 0.46 ± 0.09 and 0.52 ± 0.11 in patients with ischemic and idiopathic dilated cardiomyopathies, respectively. The change in the above ratio resulted from the fact that β_1_-AR in the epicardium remained relatively stable while β_1_-AR in the endocardium was significantly reduced. This suggests that β_1_-AR exhibits significant heterogeneity in transmural distribution in cardiac disease [24].

**Table 1:** Different sample sources and biopsy sites used for investigating β_1_-AR Expression.

Sample Sources	Biopsy site	β_1_-AR expression	Group		Value or ratio	References
Human	Left ventricle	Protein (fmol/mg)	Non-failing		88 ± 7.3	[25]
			Failing		43.2 ± 3.41	
Human	Ventricular tissue from females	Protein (fmol/mg)	Age 15.3 ± 5.5 years		86.6 ± 25.5	[22]
			Age 50.8 ± 9.1 years		49.8 ± 10.3	
Human	Ventricular tissue from males	Protein (fmol/mg)	Age 15.9 ± 4.7 years		75.1	[22]
			Age 49.4 ± 7.5 years		58.8	
Human	Left ventricle	Protein (fmol/mg)	Non-failing		73 ± 6.1	[27, 28, 182]
			Failing		35 ± 6.7	
Human	Right atrial appendage	mRNA	No MI		2.7	[177]
			MI		4.0	
Rats	Ventricular tissue	mRNA	Aged/Young		0.3	[21]
Rats	Left ventricle	Protein (fmol/mg)	4 weeks	Sham	68 ± 3.3	[142]
				PO	70 ± 3.3	
				Sham	62 ± 3.7	
				VO	113 ± 9.4	
			24 weeks	Sham	60 ± 3.4	
				PO	45 ± 2.8	
				Sham	68 ± 4.5	
				VO	47 ± 3.6	
Rats	Heart	mRNA (%)	Sham		1	[178]
			HF		≈ 0.55	
Rats	Heart	Protein (%)	Sham		1	[178]
			HF		≈ 0.66	

This table presents the expression levels of β_1_-AR in the heart under different conditions. Groups were established based on heart samples, which were obtained from different anatomical regions of various species, including humans, mice and rats. The results suggested that in the context of cardiac injury, both the mRNA and protein levels of β_1-_AR are reduced.

*MI* Myocardial Infarction, *Sal* Saline, *CM* Cardiomyopathy; Aged: this group consisted of 20–22-month male rats; Young: this group consisted of 3–4-month male rats, *PO* Pressure Overload, *VO* Volume, Overload, HF Heart Failures.

**Fig. 1:**
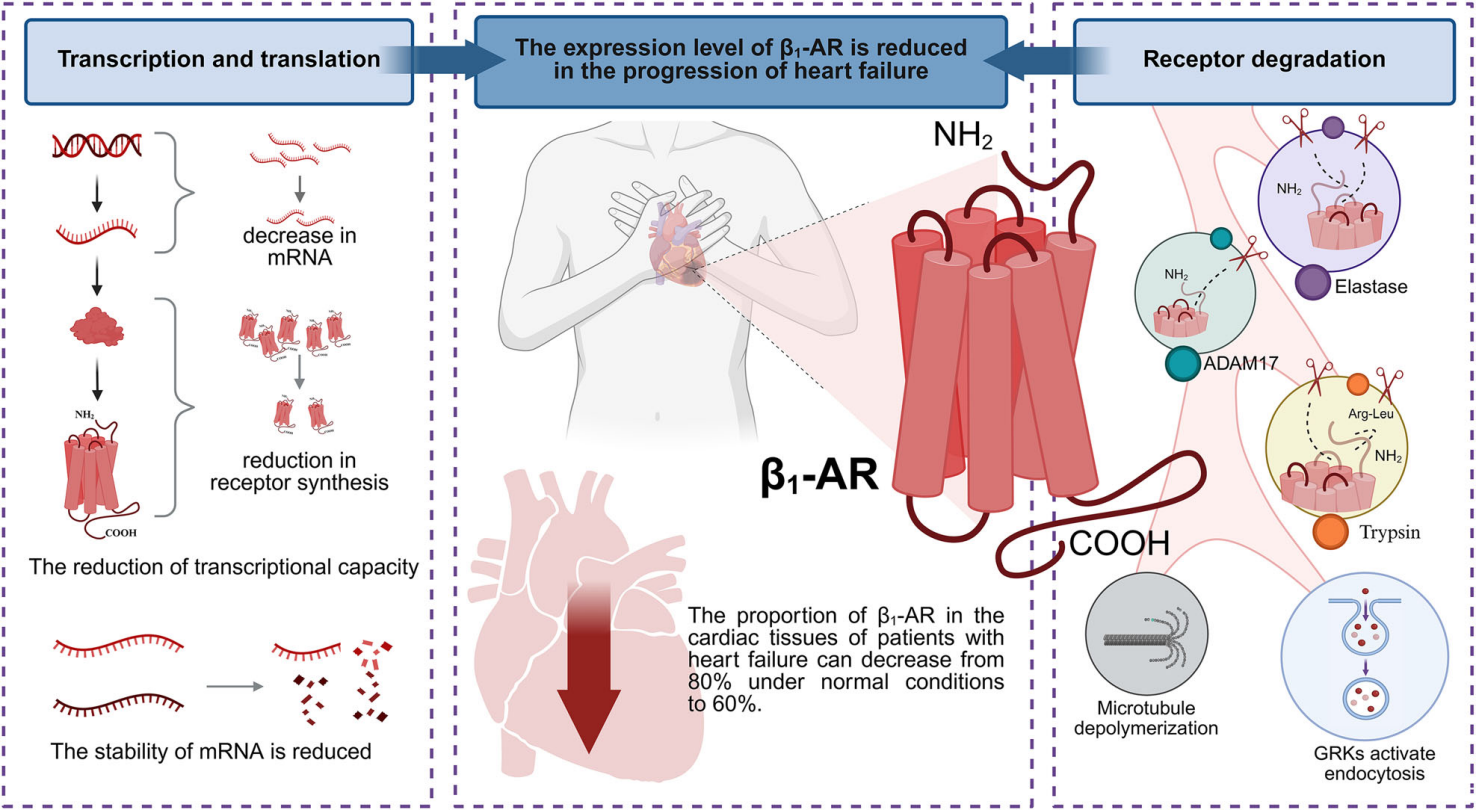
Causes of decreased β_1_-AR expression in heart failure. Reduced gene transcription capacity and enhanced protein degradation both lead to the downregulation of β_1_-AR expression. (Created with BioRender.com).

Heart failure is the end stage of many cardiovascular diseases. The percentage of β_1_-AR in cardiac tissues of heart failure patients can be reduced from 80% to 60% of normal [25, 26]. To elucidate the reasons for the reduced β_1_-AR expression in heart failure, existing studies have provided explanations in terms of both gene transcription and receptor degradation **(**Fig. 1**)**.

In myocardial tissues of heart failure patients, both messenger ribonucleic acid (mRNA) and protein contents of β_1_-AR were reduced. Among them, the mRNA content of β_1_-AR was reduced by about 50% [27] **(**Table 1**)**. Protein expression of β_1_-AR decreased from 73 ± 6.1 fmol/mg in normal heart samples to 35 ± 6.7 fmol/mg in heart failure samples [28] **(**Table 1**)**. This result suggests that the gene transcription capacity of the β_1_-AR is reduced in heart failure, leading to decreased receptor synthesis [28]. However, it has also been pointed out that the decrease in β_1_-AR expression in heart failure is not due to a decrease in transcriptional capacity, leading to reduced receptor expression. It is caused by decreased stability of β_1_-AR mRNA, making it more susceptible to degradation, ultimately resulting in a reduction in β_1_-AR expression [3].

Degradation of the receptor is also an important factor contributing to the reduction of β_1_-AR expression. It was found that β_1_-AR exposed to the membrane surface of cardiomyocytes can be cleaved by elastase. Elastase is an inflammatory protease released by activated neutrophils that accumulates at sites of cardiac injury or inflammatio,n thereby hydrolyzing β_1_-AR [29, 30]. In addition to elastase, a disintegrin and metalloproteinase 17 (ADAM17) reduces the amount of β_1_-AR by cleaving the N-terminal specific position of β_1_-AR [31, 32]. ADAM17, a redox-sensitive enzyme, shows increased expression in the heart tissue of myocarditis and dilated cardiomyopathy [33]. Trypsin can also cleave β_1_-AR in cells. In Chinese hamster ovary (CHO)-Pro5 cells, the full-length β_1_-AR NH_2_-terminus is cleaved specifically at R^31^ ↓ L^32^. In cardiomyocytes, trypsin cleaves β_1_-AR, resulting in the formation of ~40-kDa NH_2_-terminal and ~30-kDa COOH-terminal fragments [34]. Moreover, G protein-coupled receptor kinases (GRKs) in the heart activate the endocytosis mechanism by phosphorylating β_1_-AR. With the assistance of β-arrestin, the receptor is endocytosed into the cytosol and enters the lysosome for degradation. The endocytosis of β_1_-AR is a complex process, which we will describe in the context of the signaling pathway of β_1_-AR in this paper. Analysis of left ventricular myocardial samples from patients with heart failure revealed significantly increased expression of GRK2 and GRK5. This suggests increased endocytosis of β_1_-AR in heart failure [35]. Recent studies have shown that lysosomal leucine aminopeptidase (LyLAP) is highly expressed in pancreatic ductal adenocarcinoma (PDA) cells with active endocytosis. Through hydrolysis of hydrophobic peptides, LyLAP preserves lysosomal membrane integrity essential for membrane protein catabolism. When LyLAP is deficient, the hydrophobic peptide segments of the undegraded membrane proteins disrupt the lysosomal membrane, leading to lysosomal dysfunction and subsequently triggering cell death [36]. During chronic heart failure, does increased LyLAP activity in cardiomyocyte lysosomes promote enhanced β_1_-AR degradation? This question remains to be further investigated. Furthermore, although microtubule depolymerization also reduces cell-surface β_1_-AR levels, its effect is comparatively modest [37].

It can be seen that the downregulation of β_1_-AR in heart failure results from the convergence of multiple pathological factors. This β_1_-AR suppression represents a compensatory protective mechanism to mitigate receptor-mediated cardiotoxicity. While this adaptive response may confer transient cardioprotection, it ultimately heralds a more severe disease trajectory.

### The differential distribution of β_1_-AR in cardiomyocytes

**Fig. 2:**
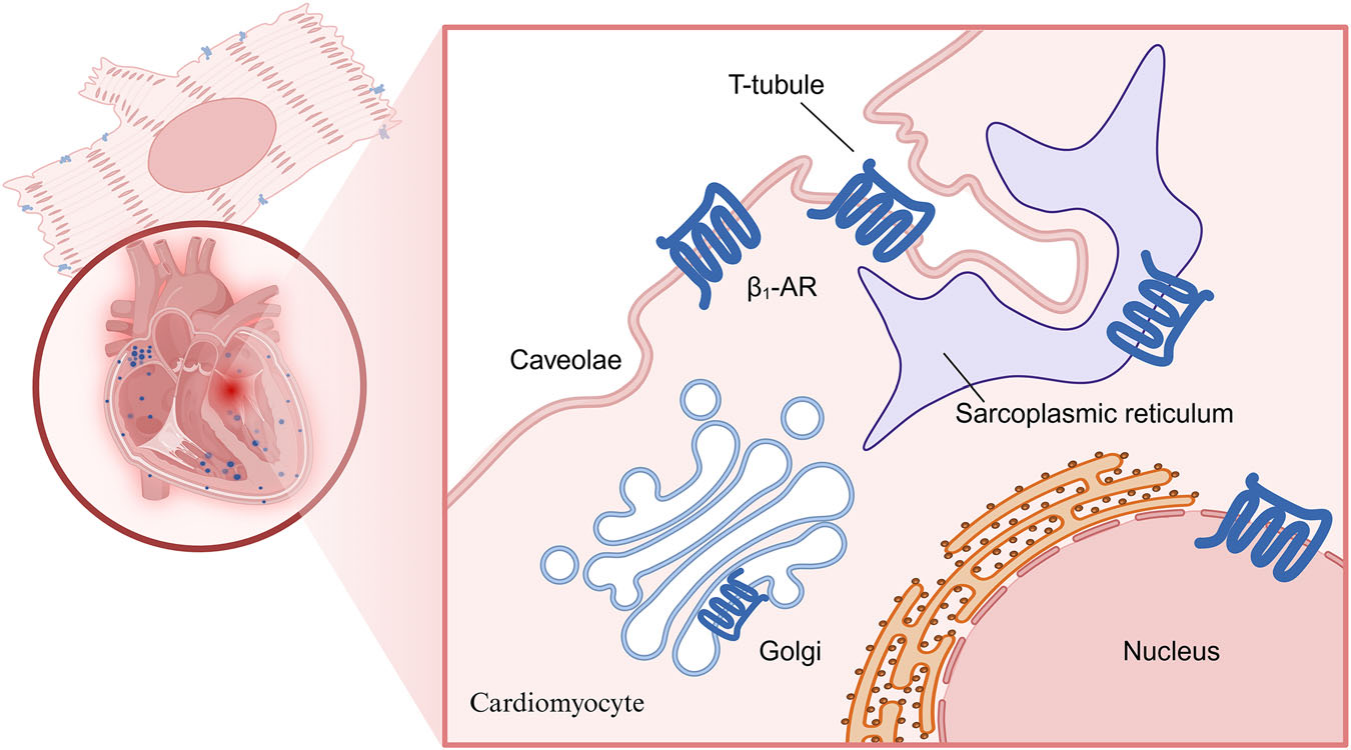
Distribution of β_1_-AR in the heart and cardiomyocytes. The distribution of β_1_-AR in the heart is mostly concentrated around the sinus node. In cardiomyocytes, β_1_-ARs localize to structures with double-membrane architectures, including T-tubules, SR, Golgi apparatus, and the nuclear envelope. (Created with BioRender.com).

β_1_-AR, as a transmembrane receptor, exhibits not only region-specific distribution patterns across cardiac compartments but also distinct spatial organization within cardiomyocyte membrane microdomains **(**Fig. 2**)**.

β_1_-AR is widely found in the cytoplasmic membrane and “T-tubular network” [38]. In the cytoplasmic membrane, β_1_-AR is concentrated in “non-caveolar cell surface membranes and internal membranes”, while only a very small amount of β_1_-AR is found in “caveolae and lipid rafts” [39, 40]. The “T-tubular network” forms specialized membrane contacts between the sarcolemma and sarcoplasmic reticulum (SR), creating microdomains where β_1_-AR orchestrates rapid transduction of sympathetic nervous signals to the cardiomyocyte.

Beyond its plasmalemmal localization, β_1_-AR exhibits distinct membrane compartmentalization across multiple intracellular organelles. Research identifies that there is functional β_1_-AR within the SR. Activation of these SR-localized β_1_-AR promotes cardiac relaxation via protein kinase A (PKA) dependent phosphorylation of phospholamban (PLN/PLB) [41]. The membrane-impermeant β_1_-AR antagonist sotalol selectively blocks plasmalemmal β_1_-AR signaling, attenuating myocardial contractility without affecting PLN phosphorylation or cardiac relaxation. The membrane-permeable β_1_-AR antagonist propranolol concomitantly inhibits both plasmalemmal and SR-localized β_1_-AR populations, thereby suppressing the phosphorylation of PLN and impairing both cardiac contractility and relaxation [42]. In a mouse model of isoproterenol (ISO) induced heart failure, the association of β_1_-AR with calcium pumps on the SR was enhanced when β_1_-AR was decreased on the cytoplasmic membrane. This phenomenon may result from either: β_1_-AR endocytosis followed by relocation to the SR or aggregation of newly synthesized β_1_-AR in the SR [43]. In addition to the SR, β_1_-AR is also present in the Golgi apparatus, where it can likewise be activated. Lipophilic ligands, such as dobutamine, passively diffuse across the plasma membrane to activate Golgi-localized β_1_-AR. Hydrophilic ligands, such as norepinephrine, rely on the organic cation transporter 3 (OCT3) located on the cell membrane to activate β_1_-AR in the Golgi apparatus [44]. Furthermore, Golgi-localized β_1_-AR activates the process of phosphatidylinositol hydrolysis via phospholipase C (PLC) and promote cardiac hypertrophy. Consequently, blocking OCT3 to restrict norepinephrine entry into cells or blocking β_1_-AR in the Golgi apparatus may serve as a prophylactic measure for endogenous ligand-dependent cardiomyocyte hypertrophy [45].

In addition to organelles, studies have also analyzed enriched cell nuclei. The results demonstrate the presence of β_1_-AR on the nuclear envelope of cardiomyocytes [46], where it exhibits biological functionality. ISO treatment activates adenylate cyclase (AC) in enriched nuclear preparations from rat hearts, with dose-dependent augmentation of cyclic adenosine monophosphate (cAMP) production [47]. In another study, it was found that treatment of isolated nuclei from rat ventricular myocytes with ISO increased the synthesis of 18S ribosomal RNA (18S rRNA). 18S rRNA is associated with protein synthesis. Meanwhile, the mRNA expression of nuclear factor κB (NFκB) was reduced. NFκB is implicated in cell proliferation and other processes [48]. This suggests that the β_1_-AR of the cell nuclear membrane may be directly involved in gene regulatory processes.

In summary, the subcellular localization of β_1_-AR in cardiomyocytes is not merely a static spatial distribution but rather dynamically engages in complex cellular signaling networks. Selective inhibition of β_1_-AR signaling in specific compartments can enable precise modulation of cardiac function.

## Protein molecular structure of β_1_-AR

The protein structure of β_1_-AR includes an extracellular NH_2_-terminal and intracellular COOH-terminal, seven-transmembrane helices (TM1-TM7), three extracellular loops (ECL1-3), and three intracellular loops (ICL1-3). Among them, the NH_2_-terminal is associated with ligand recognitio,n and the COOH-terminal is involved in downstream signaling pathways. ECL2 is the longest and most diverse loop [49]. The amino acid residues in ECL2 can affect ligand selectivity and binding affinity by directly interacting with the ligand. ECL2 is not only involved in ligand binding, but also stabilizes the ligand-binding pocket through two disulfide bonds and a sodium ion [13]. ECL3 is involved in the regulation of β_1_-AR conformational transitions and ligand recognition [50]. Each ICL contains positively charged amino acid residues that bind to negatively charged G proteins. ICL2 and ICL3 are crucial sites for binding G proteins. Specifically, ICL2 mainly enhances the binding between the receptor and G proteins [51]. ICL2 plays a pivotal “switch” role in the activation of β_1_-AR through the formation of a short α-helix parallel to the membrane surface that interacts with the highly conserved Asp138^3.49^Arg139^3.50^Tyr140^3.51^ (DRY) motif in the third transmembrane helix [13, 52]. Moreover, ICL3 is involved in the selectivity of G protein coupling [53].

The ligand-binding pocket is composed of four transmembrane helices (TM3, TM5, TM6, and TM7) and ECL2 [54]. Ligands interact with β_1_-AR through hydrogen bonds and hydrophobic interactions [55]. As an endogenous ligand, the binding of NE to β_1_-AR occurs in three steps. In the first step, NE interacts with two negatively charged amino acid residues: D217^45.51^ and D356^7.32^. In the second step, it passes through the “gate” composed of aromatic amino acid residues F218^45.52^ and F359^7.35^. In the third step, NE actually enters the orthosteric binding pocket [14]. When the ligand binds to the orthosteric site, transmembrane helices TM5 and TM6 move outward, while TM3 and TM7 shift inward, and β_1_-AR changes from a non-activated state to an activated state. Upon binding to β_1_-AR, the G protein (Gs or Gi) dissociates into α and βγ subunits, which then respectively act on downstream effectors.

When β_1_-AR binds to the Gs protein, the cytoplasmic side of TM6 is rotated outward by approximately 14 Å, TM5 is helically extended, and TM7 is moved inward by about 5 Å. In the β_1_-AR/Gs complex, the extension of the helix formed by the last four amino acids (Leu374, Tyr377, Leu379, Glu376) of the α5 helix at the C-terminus of the α subunit in Gs can increase the length of the α5 helix by 6 Å, enabling extensive contact with β_1_-AR. Concurrently, the structural domain of the α5 helix of Gs is rotated by about 96° relative to its own Ras-like GTPase domain. After rotation, the α5 helix is restricted and rotated upon contact with the Gβγ subunit. This rotation opens a tightly sealed deep groove structure within Gs, providing an exit for the release of GDP [55].

When β_1_-AR binds to the Gi protein, the α5 helix at the C-terminal of the α subunit of Gi rotates approximately 3° relative to its position in the inactive state and moves about 7 Å in the vertical direction. This results in an increased distance in the Ras-like GTPase domain of the Gα subunit, especially the α1, αG, and β6-α5 loops surrounding the GDP-binding pocket region, relative to the center of mass of the Gβγ subunit. Consequently, this leads to partial dissociation between the Ras-like GTPase domain of the Gα subunit and the Gβγ subunit, accompanied by a reduced interaction interface area and a loosened structural integrity [56].

Furthermore, upon activation, the receptor can transmit signals or mediate its own endocytosis through β-arrestin. The binding of β-arrestin to the receptor does not occur through “ligand-binding pocket”, but rather in two distinct binding modes: the “tail conformation” and the “core conformation”.

In the conformation mediated by the ICL3, β-arrestin is not close to the core of the receptor, but is attached to the C-terminal end of the receptor by a long connecting segment to form a “hanging” appearance, hence the term “tail conformation”. This conformation allows for a relatively large spatial distance between β-arrestin and the receptor, providing greater flexibility and facilitating biased agonistic signaling [57]. Deletion of the “finger loop” region in β-arrestin to construct a β-arrestin mutant reveals that the “tail conformation” can mediate receptor endocytosis and β-arrestin signaling, but is not associated with G-protein signaling desensitization [57].

The “core conformation” refers to the tight binding of β-arrestin to regions near the transmembrane helices of the receptor. In this conformation, the finger loop of β-arrestin inserts into the seven-transmembrane core domain of the GPCR. This insertion causes the outward displacement of TM3, TM5 and TM6, preventing the binding of the GPCR to the G protein and thus desensitizing the receptor [58]. When β-arrestin binds to the receptor in this manner, it can interact with the phosphorylated tail of the receptor through the NH_2_-terminal domain or directly with the core region of the receptor through the finger loop, forming an extensive action interface. The conformational changes in the internal domain of β-arrestin triggered by the “core conformation” are not only a key step in terminating the coupling of G proteins and participating in receptor endocytosis, but also contribute to stabilizing the activated state of β-arrestin, ensuring the efficiency of signaling transduction [59].

## Signaling and regulation of β_1_-AR

The signaling pathways of β_1_-AR include G protein-dependent and G protein-independent signaling pathways. The G protein-dependent pathways consist of the β_1_-AR/Gs pathway and the β_1_-AR/Gi pathway. Gs protein and Gi protein are components of G proteins, respectively mediating the activation and inhibition of AC. The G protein-independent signaling pathway is predominantly the β_1_-AR/β-arrestin signaling pathway.

### β_1_-AR and the Gs pathway

Upon activation of β_1_-AR, the activity of downstream proteases, ion channels, and transcription factors can be regulated through the β_1_-AR/Gs/AC/cAMP/PKA pathway, thereby regulating cardiomyocyte function [60, 61]. The G protein directly coupled to β_1_-AR can be dissociated into Gα and Gβγ subunits. Gα subunits can be further divided into subtypes such as Gαs, Gαi, Gαq/11, and Gα12/13. Among these, Gαs activates AC, which catalyzes the hydrolysis of ATP to cAMP.

**Fig. 3:**
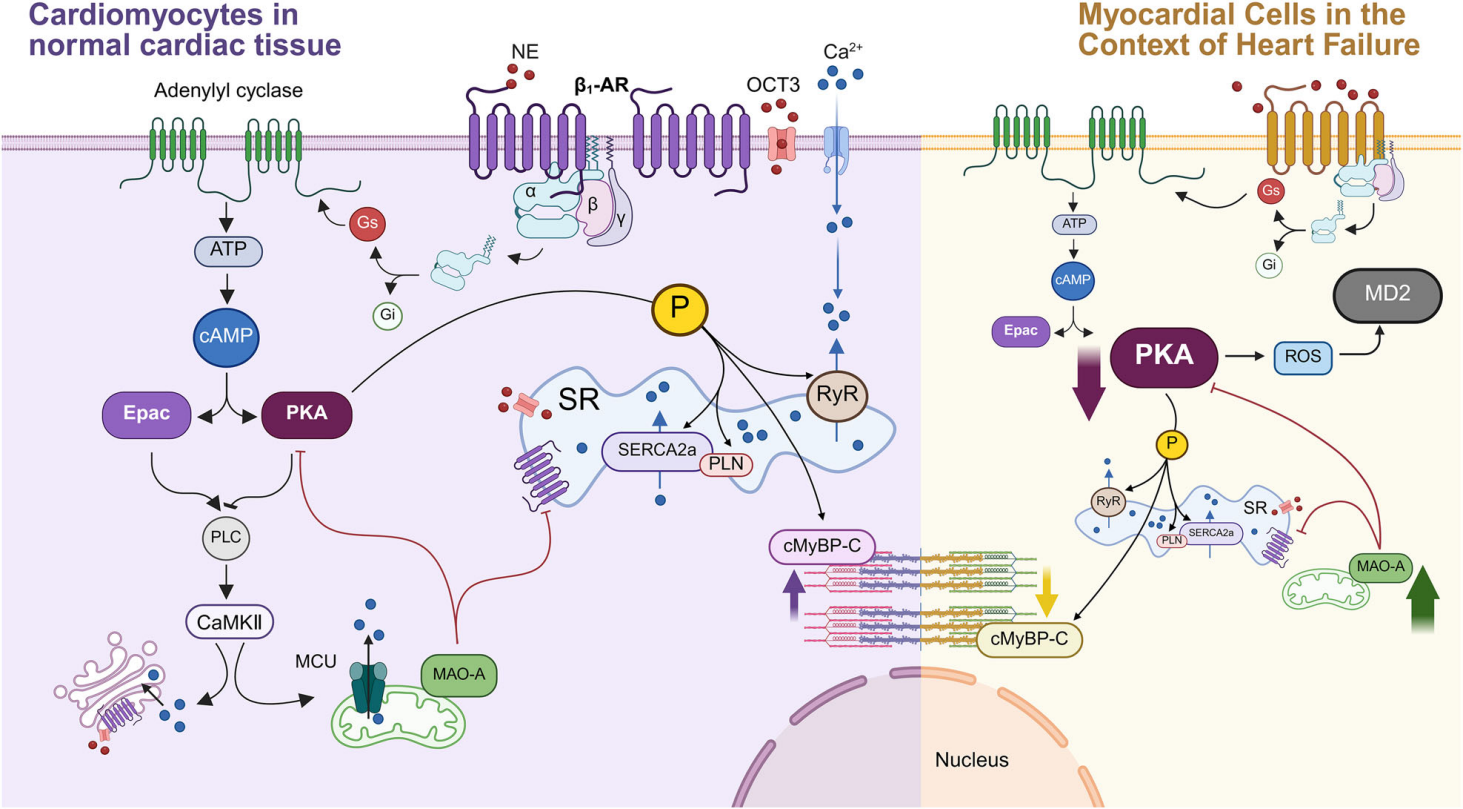
Schematic diagram of the signaling pathway after binding of β_1_-AR to Gs proteins. Upon activation of β_1_-AR by NE, the receptor couples with Gs protein, leading to dissociation into Gαs and Gβγ subunits. Subsequently, the Gαs subunit activates AC, which catalyzes the hydrolysis of ATP to cAMP. The downstream effectors of cAMP include Epac and PKA. Epac can affect the release of calcium ions from the Golgi apparatus and mitochondria through the PLC/CaMKII pathway. MAO-A on mitochondria inhibits β_1_-AR signaling and PKA activity on the SR. PKA modulates calcium ion release and reuptake at the SR by phosphorylating RyR, PLN, and SERCA2a, and enhances myocardial contractility by phosphorylating cMyBP-C. In heart failure, β_1_-AR is over-activated. PKA activity is reduced. ROS production is increased. an increased production of ROS. These molecular alterations exacerbate inflammatory responses and myocardial fibrosis. Enhanced activity of MAO-A further inhibits PKA, thus forming a vicious cycle. As a result, the activity of cMyBP-C decreases, myocardial contractility declines, and myocardial damage continues to worsen. (Created with BioRender.com).

cAMP serves as an intracellular second messenger. It binds to the regulatory subunit of PKA. PKA is an enzyme consisting of a catalytic subunit and a regulatory subunit, which acts as an effector of cAMP [62]. When the intracellular level of cAMP rises, cAMP binds to the regulatory subunit of PKA, leading to the separation of the regulatory subunit from the catalytic subunit and activation of the catalytic subunit. The catalytic subunit regulates cardiac excitation-contraction coupling by phosphorylating specific proteins [63]. This phosphorylation event potentiates the amplitude of calcium transients, enhances contractility, accelerates calcium cycling rates, and elevates pacing rates within cardiomyocytes [64] **(**Fig. 3**)**.

Cardiac excitation-contraction coupling refers to the process in which an electrical signal in the heart triggers the mechanical contraction of cardiomyocytes. It serves as the foundation for the realization of the pumping function of the heart. The action potential activates the L-type calcium channel (LTCC) located on the cardiomyocyte membrane, allowing small amounts of calcium ions to enter the cell from the extracellular space [65]. The inward flow of calcium ions not only directly participates in the contractile mechanism but also triggers the ryanodine receptor (RyR) located on the SR, causing a massive release of calcium ions stored in the SR into the cytoplasm of cardiomyocytes [66]. Increased calcium ions in the cytoplasm bind to troponin C, causing myocardial contraction. The sarcoplasmic/endoplasmic reticulum calcium ATPase isoform 2a (SERCA2a) located on the SR, opens, pumping calcium ions from the cytoplasm back to the SR for storage. As the concentration of calcium ions in the cytoplasm decreases, the cardiac muscle relaxes.

PKA increases the opening of calcium release channels by phosphorylating LTCC [67] and RyR [68], and enhances myocardial contraction by phosphorylating troponin I [69, 70]. PLN is a small-molecule protein located on the SR membrane of cardiomyocytes that modulates the activity of the SR calcium pump [71]. Phosphorylation of PLN by PKA relieves the inhibitory effect of PLN on SERCA2a, thereby facilitating the pumping of calcium ions from the cytoplasm to the SR and rapidly restoring intracellular calcium ion concentration, which shortens myocardial diastole [72]. Consequently, activation of downstream signaling molecules by the β_1_-AR/Gs/AC/cAMP/PKA pathway could enhance the excitation-contraction coupling process of cardiomyocytes, thereby increasing myocardial contractility [73].

Studies employing techniques such as Förster resonance energy transfer (FRET) have revealed that in the hearts of ISO-induced heart failure mouse models, the interactions between β_1_-AR and LTCC, as well as between β_1_-AR and RyR, are significantly diminished, leading to attenuated local PKA signaling and reduced myocardial contractility. Although the interaction between β_1_-AR and SERCA2a is enhanced, the PKA signaling at this site remains weakened [43]. This suggests that β_1_-AR, within distinct subcellular localizations and protein complexes, may be subject to specific and independent regulatory mechanisms to accommodate diverse physiological demands or pathological states.

Phosphorylation of proteins associated with the SR by PKA is regulated by monoamine oxidase A (MAO-A) [74] and organic cation transporter 3 (OCT3) [75]. MAO-A is a mitochondrial enzyme that can catabolize catecholamine substances [76]. It has been found that in heart failure, the expression of MAO-A in cardiomyocytes is upregulated, thereby inhibiting β_1_-AR/PKA signaling at the SR. Inhibition of MAO-A restored local β_1_-AR signaling at the SR and excitation-contraction coupling function of cardiomyocytes [77]. The uptake of catecholamines by cardiomyocytes requires the involvement of OCT3 [78]. In cardiomyocytes with OCT3 knockout, the phosphorylation of PLN induced by non-membrane-permeable norepinephrine was reduced, whereas the phosphorylation of PLN induced by the membrane-permeable agonist ISO and the activity of PKA were unaffected. This indicates that OCT3 is an essential component for β_1_-AR/PKA signaling in the SR [41]. Other studies similarly demonstrated that OCT3 synergizes with MAO-A to regulate β_1_-AR/PKA signaling at the cellular SR. Upon specific knockout of the MAO-A gene in the mouse heart, an increase in the phosphorylation level of proteins associated with the contractile function of cardiomyocytes on the SR was observed. Specifically, the phosphorylation of PLN was enhanced, accompanied by the activation of the β_1_-AR/PKA signaling pathway at the SR. However, following treatment with cortisol, an inhibitor of OCT3, the enhanced phosphorylation of PLN and the potentiated β_1_-AR/PKA signaling at the SR resulting from the deficiency of MAO-A can be suppressed [75].

Additionally, PKA can also phosphorylate cardiac myosin binding protein-C (cMyBP-C) in cardiomyocytes. cMyBP-C modulates cardiac muscle contraction through interactions with actin, myosin, and other contractile proteins [79]. When PKA phosphorylates cMyBP-C, the inhibitory effect of cMyBP-C on the myosin head is weakened. This leads to an accelerated cross-bridge cycle, thereby increasing the contractile force of cardiomyocytes. Moreover, the phosphorylated cMyBP-C promotes the dissociation of myosin from actin, which contributes to the acceleration of the transition of cardiomyocytes from the systolic state to the diastolic state. This is essential to maintain the effective pumping function of the heart at higher heart rates [80, 81].

In addition to PKA, the effectors of cAMP also include the Exchange protein directly activated by cAMP (Epac) [73]. Epac phosphorylates downstream proteins [82, 83], including PLN and RyR, via PLC and Ca^2+^/calmodulin-dependent protein kinase II (CaMKII), thereby affecting calcium release and calcium homeostasis of the SR [84, 85].

It was found that activation of CaMKII upregulated the expression of mitochondrial calcium uniporter (MCU). This upregulation enhanced mitochondrial calcium uptake, thereby maintaining intracellular calcium homeostasis and energy metabolism balance. As a result, it alleviated the cardiac dysfunction and remodeling caused by pressure overload [86]. The Golgi apparatus also possesses the ability to store and regulate calcium ions [87]. It has been verified that, upon stimulation of β_1_-AR, the Golgi apparatus can release calcium ions through the activation of Epac and CaMKII [88]. Thus, rectifying intracellular calcium ion cycling can improve myocardial contractility, which represents a crucial strategy for the prevention and treatment of heart failure [89].

Myeloid differentiation factor 2 (MD2) is a key protein in endotoxin-induced inflammation that mediates cardiac inflammatory damage, resulting in inflammatory heart failure [90]. ISO was found to activate MD2 in cardiomyocytes through the β_1_-AR/Gs/AC/PKA/reactive oxygen species (ROS) signaling axis. Blocking the β_1_-AR/Gs/AC/PKA/ROS signaling axis mitigates ISO-induced inflammatory response, cardiomyocyte hypertrophy, and myocardial fibrosis [91]. Yes-associated protein (YAP) is an important effector molecule in the Hippo signaling pathway. It promotes cell growth and proliferation by activating target genes through translocation into the nucleus [92]. Blockade of the β_1_-AR/Gs pathway by the β_1_-AR blocker metoprolol enhances cardiomyocyte proliferation, promotes cardiac regeneration, and improves cardiac function by activating YAP [93]. Recent studies have found that β_1_-AR reduces the expression of N6-methyladenosine (m6A). This leads to a decrease in the m6A modification level of Yap mRNA, resulting in reduced stability of Yap mRNA and decreased expression of the YAP protein. Ultimately, it inhibits cardiomyocyte proliferation and impedes cardiac regeneration [94].

### β_1_-AR and the Gi pathway

Early findings on the signaling pathway of β_1_-AR suggested that β_1_-AR couples exclusively to Gs proteins, but not to Gi proteins [95, 96]. However, as research has progressed, an increasing number of findings have fully convincing demonstrated that β_1_-AR can also bind to the Gi protein. This was because the C-terminus end of β_1_-AR contains a PDZ-binding motif. This motif specifically binds to the PDZ domain of postsynaptic density protein 95 (PSD-95), which in turn anchors β_1_-AR to the cell membrane surface and prevents its coupling with Gi protein [97]. When researchers altered the binding motif of the PDZ domain of β_1_-AR in mouse cardiomyocytes by swapping the C-termini of β_2_-AR and β_1_-AR, they found that the β_1_-AR carrying the C-terminus of β_2_-AR could both increase the myocardial beating frequency in response to ISO stimulation at the initial stage of the experiment and decrease the heart rate at the end of the experiment. Meanwhile, this phenomenon was blocked by the Gi protein inhibitor pertussis toxin (PTX). This suggests that altering the PDZ binding motif of β_1_-AR can enable it to couple with the Gi protein, leading to the occurrence of a biphasic effect. Additional studies have demonstrated that PTX inhibits the activation of extracellular signal-regulated kinases (ERK) that is mediated by β_1_-AR in Chinese hamster ovary cells (CHO cells). This indicates the involvement of Gi protein [98].

Through co-immunoprecipitation and proximity ligation experiments, researchers discovered that carvedilol, as a blocker of β_1_-AR, led to the formation of interactions between β_1_-AR and Gi, whereas NE and ISO did not produce similar effects. This is direct evidence that the β_1_-AR binds Gi, making it clear that β_1_-AR has the capability to bind to Gi. This study also found that the use of PTX inhibited carvedilol-induced ERK phosphorylation [99]. Further analysis of the β_1_-AR/Gi signaling pathway revealed that carvedilol activated the signaling pathway consisting of β_1_-AR/β-arrestin/epidermal growth factor receptor (EGFR)/ERK [100]. Activation of EGFR provides cardioprotection, thereby counteracting the cardiotoxicity of catecholamines [101]. EGFR internalization and ERK phosphorylation stimulated by carvedilol were completely blocked when β-arrestin or Gi was knocked down [99]. This suggests that carvedilol specifically activates the β_1_-AR/Gi/β-arrestin/EGFR/ERK signaling pathway to exert cardioprotective effects.

**Fig. 4:**
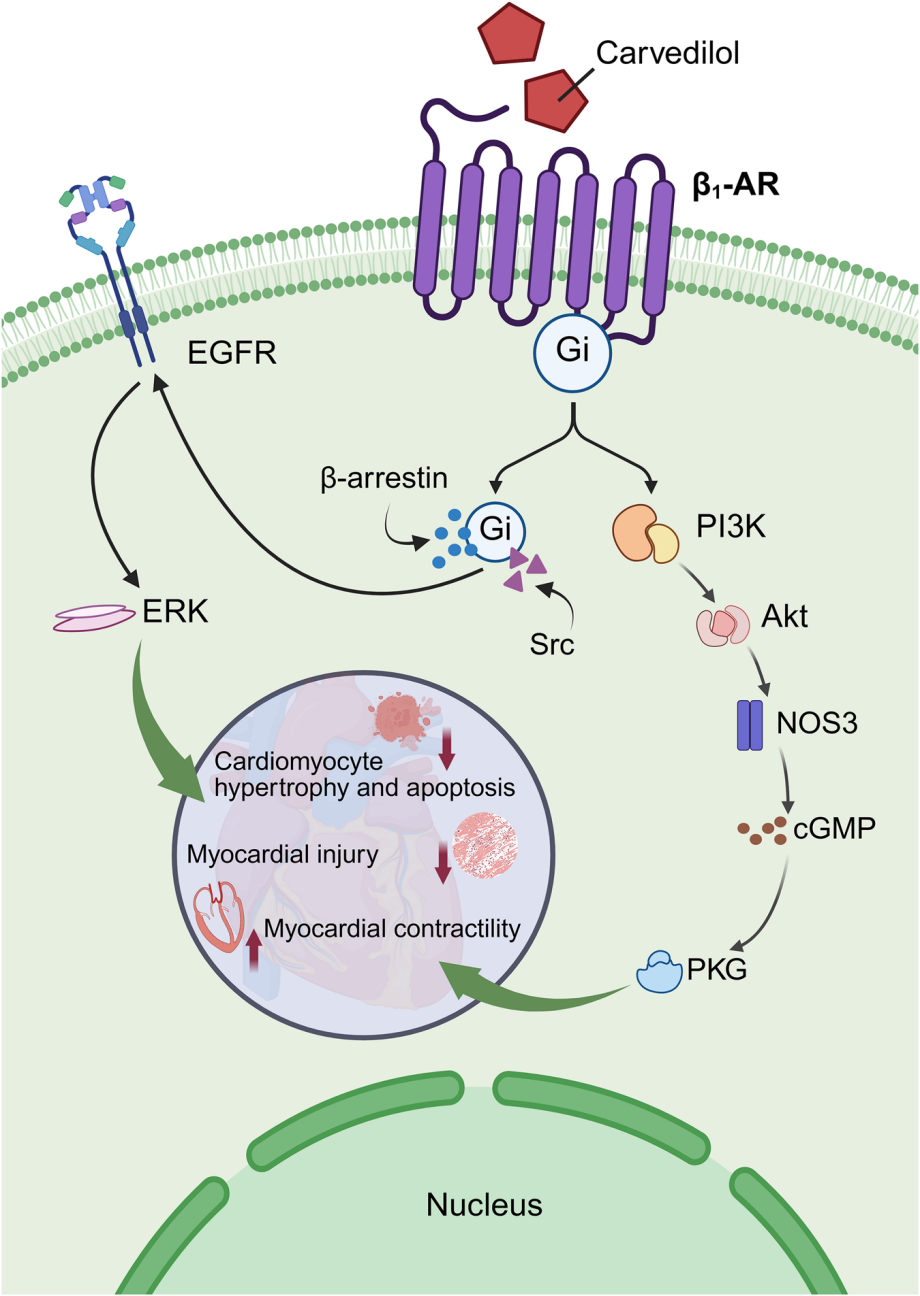
β_1_-AR couples with Gi proteins in the presence of carvedilol. There are two signaling pathways after binding of β_1_-AR to Gi protein: 1) β-arrestin/EGFR/ERK pathway; 2) β_1_-AR/Gi/PI3K/Akt/NOS3/cGMP/PKG pathway. Both pathways exert cardioprotective effects. (Created with BioRender.com).

Additionally, carvedilol activates another β_1_-AR signaling pathway: the β_1_-AR/Gi/phosphoinositide 3-kinase (PI3K)/protein kinase B (Akt/PKB)/nitric oxide synthase 3 (NOS3)/cyclic guanosine monophosphate (cGMP)/protein kinase G (PKG) pathway. Activation of this pathway enhances myocardial contractility without significantly increasing intracellular calcium ions, avoids myocardial injury caused by calcium overload, reduces cardiomyocyte hypertrophy and apoptosis, and improves both cardiac systolic and diastolic function [102] **(**Fig. 4**)**.

### β_1_-AR and the β-arrestin pathway

**Fig. 5:**
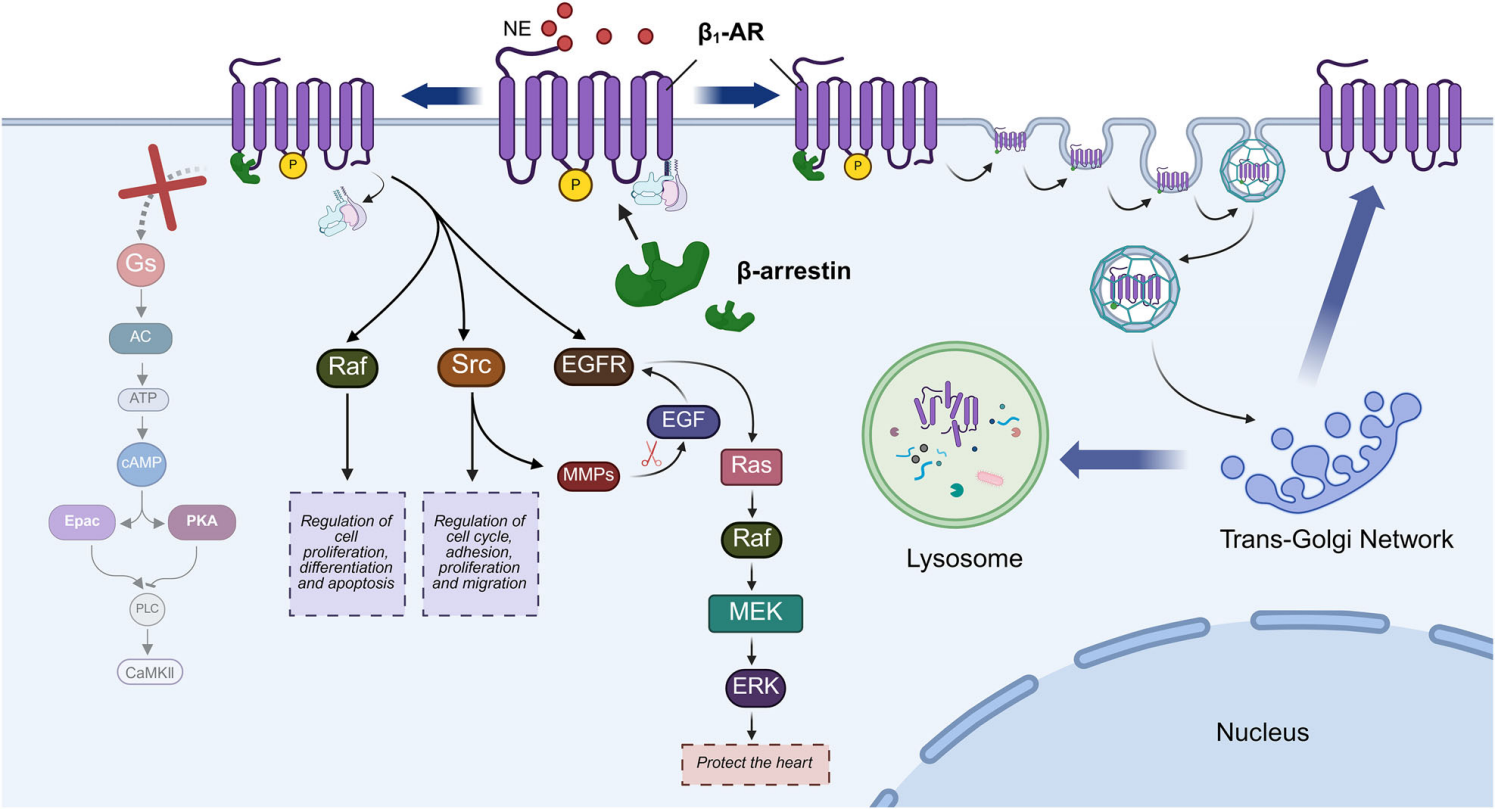
Schematic representation of the effects following the binding of β_1_-AR to β-arrestin. Phosphorylated β_1_-AR dissociates G proteins by recruiting β-arrestin. Subsequently, β_1_-AR no longer transduces signals through the AC/Gs/cAMP signaling pathway, which is termed “desensitization”. The desensitized β_1_-AR can still recruit Raf, Src, and EGFR under the action of β-arrestin, thereby activating downstream signaling pathways and exerting protective effects on the heart. Additionally, β-arrestin also mediates the endocytosis of β_1_-AR. Facilitated by the TGN, β_1_-AR can either be routed to the lysosomes for degradation or be recycled back to the cell membrane to resume its function. The binding of β-arrestin to β_1_-AR prevents overactivation of β_1_-AR. Through endocytosis, this interaction can also maintain a relatively constant number of β_1_-AR receptors on the cell membrane. (Created with BioRender.com).

β-arrestin is an adaptor protein that regulates the signaling and trafficking of GPCRs [103, 104]. Binding of β_1_-AR to β-arrestin produces two effects: desensitization of β_1_-AR and endocytosis of β_1_-AR [105]. Desensitization refers to the process in which β_1_-AR binds to β-arrestin and uncouples from G proteins to terminate β_1_-AR signaling [106]. Endocytosis refers to the process by which receptors internalize into clathrin-coated vesicles and enter the cell. Subsequently, these receptors are either transported to lysosomes for degradation or recycled to the plasma membrane to resume their functions [107]. β_1_-AR phosphorylated by GRKs recruits β-arrestin, facilitating the decoupling of the receptor from G proteins and the desensitization to prevent overactivation of the receptor. β-arrestin, in turn, can determine whether β_1_-AR is internalized [108] by recruiting adaptor proteins such as clathrin [109], adaptor protein 2 (AP2) [110], and guanine nucleotide exchange factor (GEFs) [111]. The phosphorylation pattern of the receptor and the binding of β-arrestin play a pivotal role in intracellular transport, thus determining the ultimate fate of β_1_-AR [107, 112] **(**Fig. 5**)**.

Although the desensitized β_1_-AR no longer transmits information through G protein, it continues to mediate signaling through molecules such as rapidly accelerated fibrosarcoma (Raf) [113], proto-oncogene kinase Src [114] and EGFR [115] after binding to β-arrestin. Raf is a class of mitogen-activated protein kinases that function in intracellular signaling and are involved in the regulation of processes such as cell proliferation, differentiation and apoptosis. Src is involved in the regulation of the cell cycle, adhesion, proliferation and migration [116], and is activated upon receptor desensitization [114]. Activation of Src promotes the activation of Akt [114], a key molecule necessary to mediate cardiac hypertrophy, and activates matrix metalloproteinases (MMPs). MMPs cleave and release the membrane-bound precursor forms of epidermal growth factor (EGF)-like ligands. The released EGF-like ligand then activates EGFR [115]. Activated EGFR catalyzes ERK1/2 phosphorylation through the rat sarcoma viral oncogene homolog (Ras)/Raf/mitogen-activated protein kinase (MEK) cascade reaction [117, 118], thereby antagonizing the stimulation of catecholamine and protecting the heart [101].

Following endocytosis, β_1_-AR can be directly transported to lysosomes for degradation, thereby completely terminating signaling [119]. Alternatively, a portion of β_1_-AR can be recycled back to the cell membrane, where it can re-engage in signal transduction and continue to exert its function [101, 115]. The key determinant of whether β_1_-AR is degraded or continues to function is the “Trans-Golgi Network (TGN)” [120]. TGN also determines the fate of various receptor proteins, including the G-protein-coupled estrogen receptor (GPER) [121], the growth inhibitor type 2 A receptor [122, 123], and the chemokine receptor 5 (CCR5) [124]. However, the mechanisms by which TGN regulates the fate of these proteins remain unclear.

In addition, it was found that even when clathrin-mediated endocytosis is inhibited, β-arrestin can still accumulate in clathrin-coated pits on the cell membrane and activate ERK1/2 through a “long-range activation” mechanism [125]. This indicates that β-arrestin can transmit signals through other mechanisms, although the specific details remain to be elucidated.

As a β_1_-AR blocker, carvedilol not only biases the activation of β_1_-AR towards Gi proteins, but also promotes the binding between β_1_-AR and β-arrestin. Moreover, there exists a synergistic interaction between Gi and β-arrestin, which is relevant to the signaling regulation when carvedilol acts on β_1_-AR [126]. Studies have revealed that carvedilol, following the activation of β_1_-AR, promotes signaling through the β-arrestin pathway during Dicer ribonuclease-mediated microRNA maturation, enhancing the ability of the heart to resist injury [127].

## The association between β_1_-AR and cardiovascular diseases

Pathological changes in the heart are the basis of cardiovascular diseases. The β_1_-AR is precisely closely involved in it, such as increased cardiomyocyte apoptosis [128, 129], activation of endoplasmic reticulum stress response [130], abnormal cardiomyocyte autophagy [131, 132], mitochondrial dysfunction [133], cardiomyocyte hypertrophy [134], as well as myocardial fibrosis and myocardial tissue remodeling [135, 136]. Ultimately, these pathological changes result in cardiac systolic and diastolic dysfunction, and further progress to severe cardiovascular diseases, including myocardial infarction and heart failure. Therefore, maintaining the steady-state of normal β_1_-AR function is of great significance for preserving the physiological functions of the heart.

### β_1_-AR and dilated cardiomyopathy

Dilated cardiomyopathy is defined as a condition characterized by the degeneration, hypertrophy, and necrosis of cardiomyocytes, which lead to the dilation of the left ventricle or both ventricles and systolic dysfunction. It is also accompanied by myocardial hypertrophy and reduced ventricular systolic function [137]. In dilated cardiomyopathy, a large number of cardiomyocytes undergo hypertrophy, and myocardial tissue exhibits hypertrophic remodeling to adapt to the increased preload and afterload [138, 139].

**Fig. 6:**
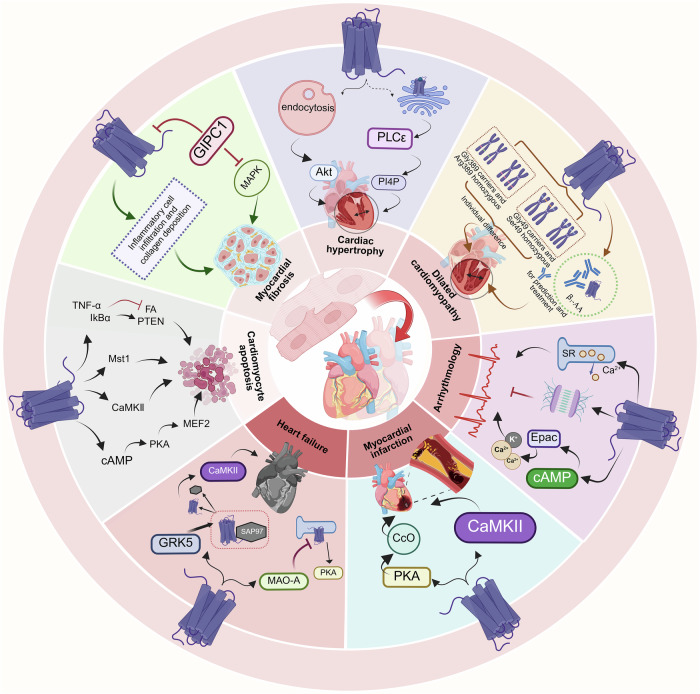
Schematic illustration of the association of β_1_-AR with different heart diseases. From cardiomyocytes apoptosis to heart failure, β_1_-AR affects cardiac diseases at different stages through distinct mechanisms. (Created with BioRender.com).

β_1_-AR induces cardiac hypertrophy predominantly through the endocytic mechanism [140]. Inhibiting various steps of the β_1_-AR endocytosis process by using the β_1_-AR blocker betaxolol, the GRK inhibitor βARK-CK, a dominant negative mutant to suppress β-arrestin, and the Src inhibitor PPI can reverse β_1_-AR-induced cardiomyocyte hypertrophy [141]. This suggests that the endocytosis of β_1_-AR is one of the causes leading to cardiac hypertrophy. Additionally, endocytosis of β_1_-AR activates Akt, which can further promote β_1_-AR-induced cardiac hypertrophy [140]. Moreover, another study has demonstrated that β_1_-AR on the Golgi apparatus can regulate cardiac hypertrophy by triggering the hydrolysis of phosphatidylinositol-4-phosphate (PI4P) through phospholipase C epsilon (PLCε). Blocking β_1_-AR on the Golgi apparatus can inhibit NE-induced cardiac hypertrophy [45] **(**Fig. 6**)**.

The signaling of β_1_-AR is related to the stages (early compensation or late decompensation) and types (pressure or volume overload) of cardiac hypertrophy [142]. In two rat models of cardiac hypertrophy induced by pressure overload and volume overload, the density of β_1_-AR **(**Table 1**)**, intracellular calcium ion concentration and AC activity were measured during the early compensatory stage (4 weeks) and late decompensatory stage (24 weeks) of cardiac hypertrophy. The results showed that all of the above indices increased at the early stage of volume overload, whereas no significant changes were observed in these indices during the early stage of pressure overload. Cardiac function remained unchanged during the early phase of volume overload, while it significantly decreased during the early stage of pressure overload. In both volumes overload-induced and late-stage pressure overload-induced cardiac hypertrophy, the above indices decreased significantly. This suggests that β_1_-AR can exert its effects through distinct signaling mechanisms in different types of cardiac hypertrophy.

Furthermore, in cardiac hypertrophy, the reduced responsiveness of β_1_-AR is not a universal phenomenon among cardiomyocytes, but rather occurs in cardiomyocytes expressing beta-myosin heavy chain (β-MHC) [143]. Cardiomyocytes were isolated after inducing cardiac hypertrophy in mice using transverse aortic constriction (TAC). Compared with β-MHC-negative cardiomyocytes, β-MHC-positive cardiomyocytes exhibited a significant reduction in percentage of cell shortening, relaxation rate, and shortening rate upon stimulation with ISO.

The genetic polymorphisms of β_1_-AR are involved in the pathogenesis of dilated cardiomyopathy [144] **(**Fig. 6**)**. In dilated cardiomyopathy, the mRNA abundance of β_1_-AR in myocardial tissue is significantly reduced [27]. In a study of the β_1_-adrenergic receptor gene signaling network (β_1_-GSN), an extensive downstream gene network of β_1_-AR has been identified. This network is associated with pathological remodeling of the left ventricle in dilated cardiomyopathy, in which metabolic genes (19.3% of the genes, of which 81% were upregulated) are dominant [145]. When analyzing the polymorphic variants Ser49Gly (rs1801252) and Gly389Arg (rs1801253) of β_1_-AR in patients with dilated cardiomyopathy, it was found that the frequency of the Gly389 allele was significantly higher in patients with dilated cardiomyopathy without ventricular tachycardia than in those with ventricular tachycardia[146]. Moreover, the Gly49 allele of the β_1_-AR was associated with a lower risk of worsening heart failure than was homozygosity for the Ser49 allele [147]. Yet another study demonstrated that patients with idiopathic dilated cardiomyopathy who carried the Gly49 allele exhibited more severe clinical symptoms and poorer prognoses (impaired cardiac function and higher rates of heart failure-related hospitalization) during follow-up [148]. Polymorphisms are present at codons 49 and 389 of β_1_-AR, resulting in a Ser49Gly (rs1801252) polymorphism and an Gly389Arg (rs1801253) polymorphism, respectively [149]. A study investigated the effects of the Ser49Gly (rs1801252) polymorphism at codon 49 as well as the Gly389Arg (rs1801253) polymorphism at codon 389 in patients with dilated cardiomyopathy [150]. The results showed that the codon 49 Ser49Gly (rs1801252) polymorphism is the dominant genetic factor influencing β-blocker response and survival in dilated cardiomyopathy. Gly49 carriers respond well to low-dose therapy while Ser49 patients require higher doses. Meanwhile, codon 389 Gly389Arg (rs1801253) has minor, context-dependent effects. This provides a basis for personalized treatment of patients with dilated cardiomyopathy.

### β_1_-AR and cardiac arrhythmias

The incidence of arrhythmia is about 0.5% [151], which triggers a variety of cardiac diseases [152]. Malignant arrhythmias such as ventricular tachycardia (VT) and ventricular fibrillation (VF) are strongly associated with sudden cardiac death (SCD) [153]. Atrial fibrillation is the most common cardiac arrhythmia and can lead to heart failure, stroke [154]. The mechanisms of arrhythmogenesis are complex, involving anatomical structures, the autonomic nervous system, ion channels, and regulatory proteins [155–157].

Although atrial fibrillation has notable genetic features, its individualized treatment has not been translated into clinical practice yet [158]. A study that analyzed the impact of the polymorphism variants Gly389Arg (rs1801253) of β_1_-AR on atrial fibrillation, revealed that the risk of postoperative arrhythmias was significantly increased among patients after cardiac surgery who were carriers of the Gly389 allele. Although atrial fibrillation has notable genetic features, its individualized treatment has not been translated into clinical practice yet [158]. The use of the third-generation β_1_-AR blocker, bucindolol, can prevent the development of atrial fibrillation and has a more pronounced effect on controlling heart rate in patients who have the Arg389Arg genotype. Patients carrying the Gly389 allele respond better to conventional β-AR blockers (e.g., metoprolol) for heart rate control [159]. In patients with supraventricular tachyarrhythmias, Arg389-homozygotes had the best results with the antiarrhythmic drug Flecainide, with good control of their heart rate. However, the efficacy is poorer in patients carrying the Gly389 allele. Patients who are Gly389-homozygous have worse heart rate control compared to those who are Gly389-heterozygous [160].

The onset of arrhythmias is not merely associated with the genetic polymorphisms of β_1_-AR, but the functional dysregulation of β_1_-AR also serves as one of the pathogenic mechanisms contributing to the development of arrhythmias.

In the hearts of mice with knockout of β-AR (including β_1_-AR, β_2_-AR, and β_3_-AR), a significant prolongation of excitatory conduction time and the refractory period was observed. Moreover, a significant reduction in atrial and ventricular arrhythmias was also noted [161].

A study investigating the role of the anti-apoptotic protein Bcl-2-assocaited athanogene 3 (BAG3) in the heart revealed that in adult left ventricular cardiomyocytes, BAG3 modulates cardiomyocyte contraction and action potential duration by interacting with β_1_-AR and LTCC, thereby reducing the risk of arrhythmias [162]. The administration of metoprolol significantly shortened the duration of norepinephrine-induced atrial fibrillation in mice. This suggests that the prolongation of atrial fibrillation duration by norepinephrine is achieved by activation of β_1_-AR. Norepinephrine not only increases calcium leakage from the SR but also promotes spontaneous calcium release, whereas metoprolol can inhibit this effect [163]. In a porcine model of ventricular fibrillation, the impact of administration of the β_1_-AR blocker esmolol during cardiopulmonary resuscitation on the recovery of calcium-circulating proteins and electrical signals was investigated. The results showed that blockade of β_1_-AR reduces ventricular fibrillation recurrence, attenuates CaMKII overactivation, and phosphorylation of RyR [164].

Desmosomal adhesion refers to intercellular adhesion mediated by desmosomes. β_1_-AR enhances cardiomyocyte adhesion through the cAMP signaling pathway. When adhesion is weakened due to desmosome disruption (e.g., tryptophan treatment), the velocity of electrical conduction slows down, and the risk of arrhythmia increases. Disruption of desmosomal proteins inhibits the cAMP signaling pathway downstream of β_1_-AR. Increasing the levels of cAMP restores the electrical conduction function in cardiomyocytes. This observation implies that the activation of the β_1_-AR/cAMP pathway can mitigate arrhythmias induced by the disruption of desmosomal structures through desmosomal adhesion [165].

Epac, an effector of cAMP, also plays a crucial role in the occurrence and development of arrhythmias. In a mouse model, β_1_-AR induces calcium leak from the SR through activation of Epac, a process dependent on phosphorylation of the RyR2-S2814 site by CaMKII [130]. It was confirmed by using the highly selective β_1_-blocker CGP-20712A, the PKA inhibitor PKI, and the highly selective Epac activator 8-pCPT, that continuous stimulation of the β_1_-AR can be mediated through the Epac-mediated Ca^2+^/calcineurin (CaN)/nuclear factor of activated T-cells (NFAT) signaling pathway, which downregulates slow current rectifier potassium current density, delays myocardial repolarization, and increases the risk of arrhythmia [166] **(**Fig. 6**)**.

### β_1_-AR and myocardial infarction

Myocardial infarction is a life-threatening disease characterized by myocardial necrosis resulting from the acute occlusion of coronary arteries. Myocardial ischemia triggers a cascade of reactions, including activation of the sympathetic nervous system and release of adrenaline [167]. Following myocardial infarction, extensive cardiomyocyte apoptosis occurs, accompanied by abnormal proliferation of cardiac fibroblasts and excessive accumulation of collagen fibers. These pathological events collectively lead to myocardial fibrosis and may even result in irreversible structural damage to the heart.

β_1_-AR is involved in the pathophysiological processes of ventricular remodeling following myocardial infarction.

Prolonged overactivation of β_1_-AR leads to programmed cardiomyocyte death [128]. After stimulating β_1_-AR with ISO in mice, the expression of the anti-apoptotic protein B-cell lymphoma 2 (Bcl-2) was significantly decreased in cardiac tissues. In contrast, the expression of the pro-apoptotic proteins Bcl-2-associated X protein (Bax) and Bcl-2 and adenovirus E1B 19-kDa-interacting protein 1 (BNIP1) was notably increased [168]. Treatment of adult rat ventricular myocytes (ARVMs) with NE or ISO increased the proportion of apoptotic cardiomyocytes by 1.72 ± 0.08-fold and 1.81 ± 0.08-fold, respectively. Terminal deoxynucleotidyl transferase dUTP nick end labeling (TUNEL) showed a 1.70 ± 0.14-fold proportion of TUNEL-positive cardiomyocytes. The apoptosis of cardiomyocytes could be reversed with CGP20712 A, a selective antagonist of β_1_-AR. This suggests that activation of β_1_-AR is a crucial factor in the induction of cardiomyocyte apoptosis [129]. Previous studies have demonstrated that β_1_-AR promotes cardiomyocyte apoptosis by inhibiting myocyte enhancer factor 2 (MEF2) activity via the cAMP/PKA pathway. Atenolol, a β_1_-AR blocker, restores MEF2 activity and thus inhibits apoptosis in cardiomyocytes [129, 169]. In addition to the cAMP/PKA pathway, β_1_-AR also induces cardiomyocyte apoptosis through CaMKII [1]. Blocking L-type calcium channels or inhibiting the activity of CaMKII prevents β_1_-AR-induced cardiomyocyte apoptosis [170]. Conversely, overexpression of CaMKII in the heart significantly increases cardiomyocyte apoptosis [170]. Moreover, β_1_-AR induces cardiomyocyte apoptosis by increasing the phosphorylation of inhibitor of kappa B alpha (IκBα) and the expression of tumor necrosis factor-alpha (TNF-α) [171], activating phosphatase and the tensin homolog (PTEN) gene, and downregulating the focal adhesion (FA) signaling pathway [172]. Mammalian sterile twenty-like kinase 1 (Mst1), a Hippo pathway mitogen-activated protein kinase (MAPK) family member, regulates apoptosis and tissue growth. Studies have shown that Mst1 reduces apoptosis in non-cardiomyocytes (such as fibroblasts, macrophages, neutrophils) and necrosis in cardiomyocytes, thereby ameliorating cardiomyopathy. Although Mst1 exerts a pivotal regulatory function during the progression of β_1_-AR-induced cardiomyopathy, the mechanism of action between Mst1 and β_1_-AR-activated signaling pathways remains unclear [173] **(**Fig. 6**)**.

Some researchers constructed myocardial infarction models using β_1_-AR knockout (β_1_-ARKO) mice, β_2_-AR knockout (β_2_-ARKO) mice, and β_1_/β_2_-AR double-KO (DKO) mice. The results showed that β_1_-AR could adversely affect the heart by activating CaMKII, which was manifested by increased apoptosis of cardiac border cells, expanded infarct area, and a decline in cardiac function indices [174]. Through experiments conducted using the isolated rabbit heart ischemia-reperfusion injury model, it was demonstrated that β_1_-AR activation leads to the phosphorylation and inactivation of cytochrome c oxidase via PKA signaling, thereby exacerbating myocardial injury **(**Fig. 6**)**. Blocking β_1_-AR could reduce the area of myocardial necrosis, reverse the decrease of cytochrome coxidase activity, and maintain the activity of mitochondrial enzyme [175]. Subcutaneous injection of ISO Isoprenaline in male Wistar rats to activate β1-AR in the heart, the results showed that myocardial lesions initiated in the subendocardial region, accompanied by the infiltration of inflammatory cells, collagen deposition, and other phenomena. The extent of lesions was proportional to the dose and duration of ISO administration. Measurement of transforming growth factor-beta 1 (TGF-β1) and brain natriuretic peptide (BNP) in serum revealed that low doses of ISO were effective in inducing changes in the above indicators [136]. Several studies have indicated that GAIP-interacting protein, C-terminus 1 (GIPC1) stabilizes the structure of the β_1_-AR by directly binding to the C-terminus of the β_1_-AR. This interaction prevents the receptor from ubiquitination and degradation, thereby maintaining the number of receptors on the cell membrane. In addition, GIPC1 exerts cardioprotective effects by inhibiting the overactivation of the mitogen-activated protein kinase (MAPK) signaling pathway. Overexpression of GIPC1 attenuates ISO-induced myocardial fibrosis and ventricular remodeling in mice [176].

Furthermore, studies have demonstrated that in the myocardial tissues of patients with myocardial infarction, the expression level of β_1_-AR mRNA is increased [177] **(**Table 1**)**. However, in the hearts of rats with myocardial infarction, the levels of both protein and mRNA of β_1_-AR are significantly downregulated [178] **(**Table 1**)**. The polymorphic variants Gly389Arg(rs1801253) of β_1_-AR were associated with myocardial infarction [179]. Among them, individuals carrying the Arg389 allele (heterozygotes and homozygous) have a significantly higher risk of developing myocardial infarction compared to those who are Gly389 homozygous [180].

### β_1_-AR and heart failure

Heart failure is a disease caused by dysfunction of the pumping function of the heart. In patients with heart failure, sympathetic overexcitation results in β_1_-AR desensitization [181]. In the myocardial tissues of patients, there is a decrease in the quantity of β_1_-AR **(**Table 1) and an increase in the amount of Gi protein [182]. The increased Gi protein inhibits cAMP formation [3, 183]. In the ISO-induced mouse model of heart failure, the protein level of β_1_-AR is decreased [43]. When β_1_-AR is specifically overexpressed in mice, cardiac contractility can be enhanced in the early stage. However, significant cardiomyocyte hypertrophy is also observed. Subsequently, mice develop heart failure with myocardial contractility dropping to 50% and ejection fraction decreasing to 20% [184]. Overexpression of β_1_-AR may improve cardiac function in the short term, yet the ultimate effect is deleterious. Similarly, in the ISO-induced mouse model of heart failure, upregulation of MAO-A limits activation of β_1_-AR/PKA signaling. The use of the MAO-A inhibitor clorgyline improves cardiac contractile function by restoring SR-localized β_1_-AR/PKA/PLN signaling [77]. Synapse-associated protein 97 (SAP97) is a scaffolding protein. It binds to the C-terminus of β_1_-AR to form the β_1_-AR-SAP97 signaling complex and regulates downstream signaling pathways [97, 185, 186]. During heart failure, the β_1_-AR-SAP97 complex is disrupted, leading to a shift of the β_1_-AR signaling pathway from the cAMP/PKA pathway to the CaMKII pathway. In cardiac-specific SAP97 knockout mice, the deletion of SAP97 promotes the binding of β_1_-AR and CaMKII and activates the CaMKII pathway. This leads to increased SR calcium leakage, cardiomyocyte apoptosis, and arrhythmias, ultimately impairing cardiac structure and function. Further studies have revealed that GRK5 promotes the dissociation of SAP97 from β_1_-AR by phosphorylating the PDZ domain of β_1_-AR, thereby activating the CaMKII pathway. In GRK5 knockout mice, CaMKII activity is inhibited and cardiac remodeling is attenuated [187]. GRK5-mediated β_1_-AR-SAP97 dissociation is a central mechanism of CaMKII overactivation in heart failure. Inhibition of GRK5 or enhancement of the binding between β_1_-AR and SAP97 improves cardiac function in heart failure patients by blocking the CaMKII signaling pathway. During the treatment of heart failure with β-blockers (e.g., metoprolol), the inhibition of the β_1_-AR signaling pathway exerts a protective effect on the heart, alleviating cardiac hypertrophy and reducing the occurrence of arrhythmias. However, molecular mechanism studies have shown that in the signaling pathway mediated by CaMKII, the expression level of the CaMKII protein is not affected by β-AR blockers. CaMKII autophosphorylation is also not affected. Moreover, the phosphorylation status of downstream target proteins, such as RyR, remains unaffected by β-AR blockers [188]. Therefore, although β-AR blockers are used in the treatment of heart failure, they are unable to suppress the pathological cardiac remodeling driven by CaMKII. Targeted inhibition of the CaMKII pathway may be a complementary strategy for the treatment of heart failure in the future **(**Fig. 6**)**.

In studies of polymorphism variants Gly389Arg (rs1801253) of β_1_-AR, patients who are Arg389 homozygous were found to have a better response to treatment with β_1_-blockers (e.g., carvedilol) and a more pronounced improvement in left ventricular function than patients who are carriers of the Gly389 allele [189]. Another study showed that patients carrying the Gly389 allele had a significantly increased risk of heart failure in East Asian populations, whereas they tended to have a reduced risk in Caucasian populations. Patients who are Arg389 homozygous uniformly exhibit a more favorable therapeutic response to selective β_1_-blockers (e.g., metoprolol), as evidenced by significant improvements in left ventricular ejection fraction and left ventricular end-systolic diameter/volume [190]. Thus, despite the association of β_1_-AR gene states with heart failure, there are racial differences in their effects. Currently, there is insufficient evidence to support their use as a basis for personalized disease treatment.

### β_1_-AA in cardiovascular diseases

β_1_-adrenergic receptor autoantibody (β_1_-AA) is an autoantibody generated against the second extracellular loop of β_1_-AR. It possesses the function of sustaining activation of β_1_-AR [191, 192].

Studies have demonstrated that β_1_-AA promotes myocardial fibrosis [193], inhibits cellular autophagy and induces cardiomyocyte apoptosis [194, 195].

β_1_-AA is also closely associated with the development and progression of multiple diseases, including dilated cardiomyopathy [196], atrial fibrillation [194, 197], and heart failure [198, 199]. In patients with dilated cardiomyopathy, the positive rate of β_1_-AA is as high as 30% [200]. After removing β_1_-AA from the peripheral blood of patients via immunoadsorption, the clinical manifestations of these patients are significantly improved [201].

The aforementioned diseases have been further validated through animal studies. Passive immunization of mice with β_1_-AA leads to significant reduction in cardiac function and myocardial remodeling [198]. Additionally, passive immunization of rabbits with β_1_-AA results in obvious arrhythmias [202]. Based on the results of the aforementioned studies, β_1_-AA exhibits pathogenicity.

The core consensus regarding its pathogenic mechanism is that β_1_-AA sustained actives of β_1_-AR [203]. Specifically, in cardiomyocytes, β_1_-AA induces a prolonged elevation of the cardiomyocyte beating frequency by sustaining β_1_-AR activation, whereas norepinephrine only causes a transient increase in cardiomyocyte beating frequency [192]. Concurrently, the intracellular cAMP level also increases persistently. Current studies propose two explanations for the sustained activation of β_1_-AR by β_1_-AA: 1) β_1_-AA stabilizes β_1_-AR in an active conformation to maintain its continuous activation [204]; 2) β_1_-AA inhibits the endocytosis of β_1_-AR, thereby enabling its sustained activation [192].

Currently, the primary therapeutic strategy for patients with β_1_-AA-positive cardiac diseases is immunoadsorption therapy [201, 203]. Additionally, specific cyclic peptides [205] or aptamers [206] are also used to specifically bind to β_1_-AA and neutralize its activity. Among these approaches, immunoadsorption has been applied in clinical practice and significantly alleviated the symptoms of patients [199]. Furthermore, biased activation of the β_2_-AR-Gi signaling pathway also inhibited the sustained activation of β_1_-AR induced by β_1_-AA [192, 198]. However, the clinical application prospects of this method still require further research.

### β_1_-AR blockers

In the treatment of cardiac diseases, pharmacological intervention has significantly reduced morbidity and mortality [207]. Among the available therapeutic approaches, β_1_-AR blockers act on the sympathetic nervous system to inhibit β_1_-AR-mediated signaling pathways, which in turn reduces heart rate and myocardial oxygen consumption, thereby improving cardiac function. Notably, β_1_-AR blockers exhibit more pronounced therapeutic effects in the treatment of heart failure and the management of cardiac arrhythmias [207].

β_1_-AR blockers are organic compounds composed of an aryloxypropanolamine side chain, with an aromatic or heteroaromatic ring attached to this side chain [208]. Although different β_1_-AR blockers vary in their substituents, they generally share a fundamental aryloxypropanolamine structure, which serves as the basis for their pharmacological activity [209]. β_1_-AR blockers bind competitively and reversibly to the endogenous ligand-binding site of β_1_-AR. Upon binding, β-blockers and β_1_-AR form a stable complex. However, the conformation of β_1_-AR has no change. By occupying the binding site of β_1_-AR, they prevent catecholamines from interacting with β_1_-AR, thereby inhibiting G protein activation, blocking AC stimulation, and reducing intracellular cAMP levels. This suppression of downstream molecule activation ultimately blocks or attenuates the positive cardiac effects mediated by the β_1_-AR signaling pathway, exerting therapeutic effects such as antiarrhythmic action, antianginal activity, and improving heart function [210].

β_1_-AR blockers are mainly categorized into three generations. First-generation blockers exhibit no selectivity towards β-AR subtypes, such as propranolol and nadolol. Second-generation selective β_1_-AR blockers include metoprolol, atenolol, bisoprolol, and landiolol. Third-generation selective β_1_-AR blockers include carvedilol, nebivolol, and labetalol [211].

The first generation of non-selective β-AR blockers not only block β_1_-AR but also β_2_-AR, and they have no specificity for either subtype. The blockade of β_2_-AR, leads to an increase in peripheral vascular resistance, bronchial contraction, inhibition of glycogenolysis, and altered lipolysis. Therefore, in clinical practice, first-generation non-selective β-AR blockers should be applied with caution or contraindicated in patients with diabetes mellitus or pulmonary diseases [207].

Second-generation selective β_1_-AR blockers exhibit specific antagonism toward β_1_-AR with only weak affinity for β_2_-AR. Therefore, they are particularly suitable for patients with heart diseases who also have pulmonary conditions [208].

Third-generation β_1_-AR blockers exert antagonistic effects not only on β_1_-AR but also α_1_-AR. They have minimal impact on glucose and lipid metabolism and possess vasodilatory properties. Consequently, their main side effect is hypotension. Clinically, they are suitable for elderly patients as well as those with cardiovascular diseases and diabetes mellitus [209, 211, 212].

**Fig. 7:**
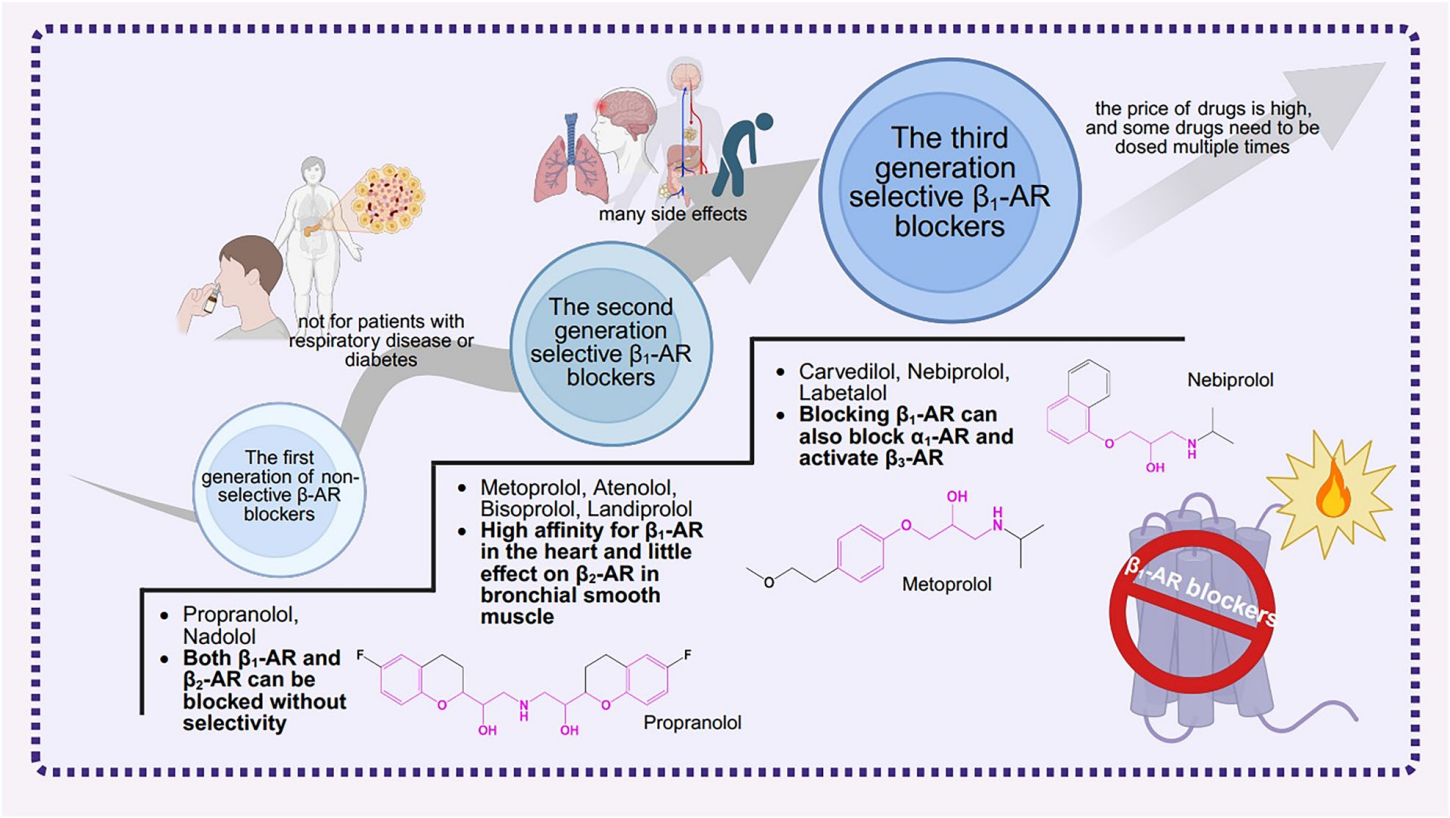
Development and differentiation of β_1_-AR blockers. The adverse effects of first-generation β-AR blockers, particularly bronchoconstriction and metabolic dysregulation, motivated the development of second-generation selective β_1_-AR blockers. Subsequently, third-generation selective β_1_-AR blockers have been designed to possess vasodilatory properties, optimizing their hemodynamic profile. Furthermore, the structural schematic diagram of the representative drugs of three generations of β_1_-AR blockers (metoprolol, nebivolol, propranolol) is presented. The common structure marked in pink in the figure is aryloxypropanolamine. (Created with BioRender.com).

In summary, first-generation β-AR blockers have basic efficacy, such as reducing myocardial contractility and hypotensive effects. However, they are associated with more side effects. Second-generation drugs specifically block β_1_-AR, improving cardiac selectivity and drug safety more significantly. In addition to targeting β_1_-AR, third-generation drugs also act on α_1_-AR and β_3_-AR, further optimizing therapeutic effects and reducing side effects through synergistic actions of multiple targets and mechanisms [208] **(**Fig. 7**)**.

Propranolol is a first-generation non-selective β-AR blocker [213]. It reduces heart rate, cardiac output, and myocardial contractility by blocking cardiac β_1_-AR. It can effectively treat hypertension, angina pectoris, and myocardial infarction. However, it also blocks β_2_-AR, which inhibits hepatic glycogenolysis, delays the recovery of blood glucose and reduces symptoms of hypoglycemia such as palpitations and hand tremors. Therefore, it should be used with caution in patients with hypoglycemia. Blocking β_2_-AR may also cause bronchospasm. Hence, it is contraindicated in patients with asthma [214]. Although propranolol is inexpensive, it is no longer used as a preferred drug for the treatment of hypertension and other cardiovascular diseases because of its relatively numerous side effects. Clinical studies are now mostly focused on the treatment of infantile hemangiomas [215] and portal hypertension [216].

Landiolol is a second-generation β_1_-AR blocker that has been extensively investigated in recent years. It is characterized by its ultra-short-acting property and high selectivity [217]. It exerts its effects by competitively antagonizing β_1_-ARs, thereby inhibiting the action of catecholamines. This mechanism attenuates the heart rate acceleration induced by sympathetic nerve activation, which in turn helps control cardiac arrhythmias and reduces adverse impacts on cardiac function [217]. It is mostly used for the treatment of tachyarrhythmias, especially atrial fibrillation [218], sepsis-associated tachycardia [219, 220], and for preoperative heart rate control in coronary artery and other vascular interventions [221, 222]. Compared with other blockers, landiolol has a more potent chronotropic effect and a weaker hypotensive effect [223]. In recent studies, patients with supraventricular tachycardia demonstrated excellent tolerability to landiolol and had a favorable safety profile [224]. In a canine model of endotoxic shock, a low dosage of landiolol reduces heart rate without affecting hemodynamic recovery in the early stages of shock [225]. The common adverse effects of landiolol include hypotension and bradycardia. In severe cases, it may lead to complete atrioventricular block and sinus node arrest. Therefore, the blood pressure and cardiac rhythm of patients should be strictly monitored during its administration.

Metoprolol also belongs to the second generation of β_1_-AR blockers. It is commonly used as a combined medication for hypertension [226] and for adjunctive treatment of hypertrophic cardiomyopathy [227], atrial fibrillation [228], and heart failure [229]. Studies have revealed that intravenous administration of metoprolol before reperfusion in patients with acute myocardial infarction can improve left ventricular function. Moreover, the rate of readmission due to heart failure was significantly lower in patients treated with metoprolol [230]. These findings have underscored the importance of using metoprolol for early intervention in myocardial infarction to improve the prognosis. Studies have shown that whether metoprolol is administered to rats acutely via oral intake or chronically through addition to drinking water or intraperitoneal injection, it can produce cardioprotective effects. Nevertheless, metoprolol also increases the risk of ischemic stroke by inhibiting cerebrovascular β_1_-AR-mediated vasodilation [231]. Furthermore, metoprolol, although not a conventional antiarrhythmic drug, has demonstrated an effect in neonatal foals with irregular tachyarrhythmias into a normal sinus rhythm in clinical cases of treating neonatal foals [232]. Early studies have also demonstrated that metoprolol can prolong the effective atrial action potential refractory period and reduce the fibrillation frequency in a porcine model of atrial fibrillation [233]. The most common adverse effects of metoprolol are bradycardia and hypotension, which are dose-dependent.

Nebivolol, a third-generation β_1_-AR blocker, is used for the treatment of conditions such as hypertension and heart failure [234]. Nebivolol not only produces cardioprotective effects by blocking β_1_-AR, but also exhibits anti-inflammatory, antioxidant, and vascular endothelial protection effects [208]. Nebivolol is frequently used in combination with other antihypertensive drugs to treat hypertension [235, 236]. The most common adverse reactions include fatigue (4%-79%), headache (2%-24%), and bradycardia (6%-11%). Nebivolol is potentially superior for patients who are intolerant to traditional β_1_-AR blockers, such as those with asthma or chronic obstructive pulmonary disease [237, 238].

ABRQβ-006 is a novel therapeutic vaccine designed to target the β_1_-AR via an active immunization strategy. Its mechanism of action involves the chemical conjugation of ABR-006, a specific peptide derived from the second extracellular loop (ECL2) of the human β_1_-AR, to Qβ phage virus-like particles (VLPs). Following vaccination, this vaccine elicits the production of high-titer specific antibodies in the body. These antibodies bind to β_1_-ARs and exert a functional antagonistic effect, thereby inhibiting the downstream pathological signaling pathways mediated by excessive sympathetic nerve activation. In preclinical studies, ABRQβ-006 has demonstrated therapeutic potential for a variety of cardiovascular diseases. Specifically, it effectively reduces systolic blood pressure and ameliorates vascular and myocardial remodeling in hypertensive models. In pressure overload (transverse aortic constriction, TAC) and myocardial infarction (MI) models, it significantly improves cardiac function, inhibits ventricular remodeling, and attenuates myocardial fibrosis and inflammatory infiltration. Notably, its efficacy in MI models is superior to that of metoprolol. These findings indicate that ABRQβ-006 holds broad application prospects in the treatment of hypertension and heart failure [239].

## Perspectives

Over the past century, researchers have made a series of important discoveries in analyzing the molecular structure of β_1_-AR and elucidating the signaling mechanism of β_1_-AR.

Despite some achievements made in the research around β_1_-AR, there remain numerous unresolved issues that merit our attention. For instance, the traditional view holds that β_1_-AR binds to Gs protein rather than Gi protein. However, it has now been confirmed that carvedilol can induce the binding of β_1_-AR to Gi protein, enabling it to function through a signaling pathway distinct from that of Gs [99]. This finding reveals the complexity and diversity of β_1_-AR functions. Does NE in vivo also induce the binding of β_1_-AR to Gi protein? Current research has not yet provided a definitive answer.

As for Gs protein, the conformation of the β_1_-AR/Gs complex is distinct when bound to agonists of varying efficacy, including full, partial, and weak agonists [240]. Further studies showed that the full agonist-bound β_1_-AR/Gs complex was the most stable and induced the fastest activation and termination of the β_1_-AR/Gs signaling pathway. This suggests that there are differences in the efficiency with which the same receptor activates the downstream signaling pathway with different ligands bound.

In addition to Gs and Gi, there are other types of G proteins, such as Gαq/11 and Gα12/13 [241]. It remains unclear whether these aforementioned G proteins can also couple with β_1_-AR and mediate distinct signaling pathways. The actual diversity of existing G proteins is far greater than previously recognized. The human genome encodes over 20 different Gs, each exhibiting specific tissue distribution and functional characteristics [242]. Consequently, a full consideration of the structural features, expression patterns of each molecule, and their interactions with other cellular components is required to better understand the cellular signaling network of β_1_-AR.

β_1_-AR serves as a crucial therapeutic target for various heart diseases. Drugs developed targeting β_1_-AR are not only applicable in the cardiovascular system but also involved in the treatment of neurological, urological, anti-tumor, and other multifaceted diseases. Therefore, drug development against β_1_-AR still has a wide range of prospects and important significance. At present, β_1_-AR blockers have been developed to the third generation. Given that the first-generation blockers are nonselective and restrict patients with concomitant respiratory disease when used to treat cardiovascular disease, the second-generation blockers were developed. Compared to the first-generation blockers, the second-generation blockers exhibit higher selectivity for β_1_-AR and improve patient tolerance. However, they still cause more side effects such as fatigue, coldness, and fluctuations in blood glucose levels. The development of third-generation blockers aims to further optimize efficacy and reduce side effects. These drugs provide effects not only of β_1_-AR blockade, but also of vasodilation, anti-inflammation and anti-oxidation, offering more comprehensive cardiac protection. The advancement of new-generation β_1_-AR blockers necessitates a multi-faceted approach. Primarily, the mitigation of adverse effects should be prioritized through refined molecular design strategies. Secondarily, comprehensive improvements in cardiac and vascular functionality are imperative via biased activation of specific signaling pathways. Additionally, modification of the pharmacokinetic profile is essential, entailing the optimization of drug half-life, distribution, and metabolism. This modification aims to extend the action duration of drugs, decrease the dosing frequency, and improve patient compliance, thereby maximizing clinical efficacy. These efforts can help achieve a more desirable therapeutic effect.

Although this article exclusively focuses on the role of β_1_-AR in the heart and related research, the distribution and function of β_1_-AR are far from being limited to cardiac tissues. β_1_-AR is also distributed in many other organs and tissues, such as the central nervous system, kidneys, and adipose tissues, where it plays important functions. In the central nervous system, it is involved in the regulation of neurotransmitters [243, 244]. In the kidneys, it regulates renin secretion and affects water and salt metabolism [245, 246]. In adipose tissues, it governs lipolysis and is involved in energy metabolism [247]. Therefore, when using β_1_-AR blockers in the treatment of cardiac diseases, it is necessary to consider the potential effects of the drugs on β_1_-AR in non-cardiac tissues.

In recent years, with the deepening of research, the application potential of β_1_-AR blockers has extended beyond the traditional cardiovascular field, presenting broader therapeutic prospects. Non-selective β_1_-AR blockers, represented by propranolol, have demonstrated remarkable anti-tumor effects in various cancer models [248]. Therefore, the research and development strategies of β_1_-AR blockers should focus on multi-target design to achieve precision therapy while reducing the side effects of the drugs.

This review summarizes the process of scientific exploration targeting β_1_-AR, points out the distribution features of β_1_-AR in the heart, and summarizes the signaling pathways mediated by β_1_-AR under different conditions, as well as the research progress related to β_1_-AR and drug applications in various cardiac diseases. It is of great significance to deepen the biological understanding of β_1_-AR and promote the advancement of the treatment of cardiovascular diseases.

## Data Availability

No datasets were generated or analysed during the current study.
